# Beyond Neurodevelopmental Delay: *BICRA*-Related Coffin–Siris Syndrome 12 with Severe Intestinal Dysmotility and Recurrent Pneumothorax

**DOI:** 10.3390/genes17010081

**Published:** 2026-01-11

**Authors:** Hua Wang

**Affiliations:** Division of Genetics, Department of Pediatrics, School of Medicine, Loma Linda University, 11175 Campus Street, CP 11121E, Loma Linda, CA 92350, USA; huawang@llu.edu

**Keywords:** Coffin–Siris syndrome 12, *BICRA*, chromatin remodeling, gastrointestinal dysmotility, pneumothorax, long-term follow-up, expanded phenotype

## Abstract

Background: Coffin–Siris syndrome 12 (CSS12) is a recently described neurodevelopmental disorder caused by heterozygous pathogenic variants in *BICRA*, a gene encoding a core subunit of the non-canonical BAF (ncBAF) chromatin-remodeling complex. The condition is characterized by developmental delay, hypotonia, hypertrichosis, and joint laxity. However, long-term data remain limited, and systemic manifestations are incompletely defined. Case Description: We report a 22-year-old male with a de novo *BICRA* frameshift variant, *c.2479_2480delinsA* (p.Ala827Thrfs*15), previously included in the original cohort reported by Barish et al. Longitudinal follow-up revealed an expanded phenotype extending beyond neurodevelopmental features. Early findings included global developmental delay, growth hormone deficiency, short stature, and joint hypermobility. In adolescence and adulthood, he developed severe intestinal dysmotility requiring total colectomy, recurrent spontaneous pneumothoraces from bilateral apical bullous disease, and portal-vein thrombosis, representing visceral and vascular complications not previously emphasized in *BICRA*-related disorders. The identified *BICRA* variant truncates the coiled-coil domain critical for BRD9/BRD4 interaction, consistent with a loss-of-function mechanism. The patient’s systemic features suggest that *BICRA* haploinsufficiency affects not only neurodevelopmental pathways but also smooth-muscle and connective-tissue integrity. Conclusions: This case expands the phenotypic spectrum of *BICRA*-related CSS12, demonstrating that visceral and vascular involvement can occur alongside neurodevelopmental and connective-tissue features. Recognition of these broader manifestations underscores the need for lifelong multidisciplinary surveillance and contributes to understanding the diverse biological roles of the ncBAF complex in human development.

## 1. Introduction

Coffin–Siris syndrome (CSS) is an autosomal dominant genetically heterogeneous neurodevelopmental disorder characterized by developmental delay or intellectual disability, hypotonia, coarse facial features, hypertrichosis, and variable multisystem involvement. CSS belongs to the group of SWI/SNF-related intellectual disability disorders (SSRIDDs), caused by pathogenic variants in genes encoding subunits of the ATP-dependent chromatin-remodeling BAF (BRG1/BRM-associated factor) complexes [1]. These complexes regulate chromatin accessibility and gene expression during development, and their disruption leads to a broad phenotypic spectrum ranging from isolated neurodevelopmental disorders to syndromic malformations [2,3]. Pathogenic variants in several SWI/SNF complex genes—including ARID1A, ARID1B, SMARCA4, SMARCB1, SMARCE1, SOX11, and more recently, BICRA—have been implicated in CSS and related disorders [1,4].

In 2020, Barish and colleagues identified BICRA (BRD4-interacting chromatin-remodeling complex-associated protein A) as the gene responsible for a distinct subtype, now designated Coffin–Siris syndrome 12 (CSS12; OMIM #619325). *BICRA* encodes a scaffold subunit of the non-canonical BAF (ncBAF) complex, which interacts with BRD9 and BRD4 to regulate enhancer activity and transcriptional elongation [5]. Pathogenic *BICRA* variants—almost exclusively truncating or frameshift changes—result in haploinsufficiency of the ncBAF complex and produce a characteristic but variable phenotype, including developmental delay, mild-to-moderate intellectual disability, joint hypermobility, and coarse facial features. Although Barish et al. [4] described twelve individuals establishing *BICRA* as a disease-causing gene, detailed clinical information and long-term follow-up were limited. Subsequent reports have added isolated cases, further confirming the association of BICRA variants with neurodevelopmental impairment, gastrointestinal and ophthalmologic involvement, growth retardation, and craniofacial dysmorphism [6,7,8]. However, visceral and connective-tissue manifestations remain poorly characterized, and the full clinical spectrum of BICRA-related disorders is still emerging.

Here, we present the long-term clinical follow-up of an individual originally included in the Barish et al. [4] cohort, now age 22 years, harboring a de novo pathogenic BICRA frameshift variant. This extended observation demonstrates a markedly expanded phenotype, including severe gastrointestinal dysmotility requiring colectomy, recurrent spontaneous pneumothoraces, and vascular/connective-tissue complications—features not previously emphasized in BICRA-related CSS. This report underscores the importance of longitudinal follow-up to delineate the evolving phenotype and natural history of recently defined genetic disorders such as BICRA-related Coffin–Siris syndrome.

## 2. Case Description

The patient is a 22-year-old male of mixed European descent, the first child of non-consanguineous healthy parents, followed by Medical Genetics since age 10. He was born at term after an uncomplicated pregnancy and spontaneous vaginal delivery, with a birth weight of 3.2 kg and normal Apgar scores. The neonatal course was uneventful. Feeding difficulties and constipation began within the first few weeks of life, accompanied by chronic abdominal distention and poor weight gain. Developmental delays were evident in infancy: he rolled at 9 months, sat at 13 months, and walked at 18 months. Language milestones were mildly delayed, with first words at 2 years and short phrases by age 4. There was no developmental regression, and social interaction was appropriate.

During childhood, constipation progressed and became refractory to medical therapy. Imaging demonstrated marked colonic dilation consistent with functional megacolon, while rectal biopsy confirmed the presence of ganglion cells, excluding Hirschsprung disease. Between ages 11 and 13, he underwent sigmoidectomy and temporary colostomy for recurrent fecal impaction and bowel obstruction. Despite these procedures, colonic dysmotility persisted, and at age 18 he required a total colectomy. Pathologic examination showed muscular hypertrophy and neuronal hyperplasia, consistent with a functional motility disorder rather than aganglionosis. He was diagnosed with growth hormone deficiency and short stature in early childhood and received recombinant growth hormone therapy (Omnitrope) for two years, with partial improvement in growth velocity. Cognitive testing performed through his school program at age 11 demonstrated mild-to-moderate intellectual disability (full-scale IQ ≈ 75, verbal comprehension 69, working memory 86). He continued in a special-education curriculum with speech and occupational therapy support. Adolescence was notable for generalized joint hypermobility (Beighton 6/9) with recurrent knee dislocations and psoriatic arthritis, which responded well to adalimumab. At 19 years, he experienced two episodes of spontaneous pneumothorax caused by bilateral apical bullous lung disease, managed with chest-tube drainage. Additional complications included portal-vein thrombosis treated successfully with anticoagulation and mild right-ventricular dilation on echocardiography.

A first genetics evaluation in 2017 (14 years old) included chromosomal microarray and *FMR1* (Fragile X) testing, both of which were normal, as well as an intellectual-disability gene panel revealing several variants of uncertain significance (VUS) not involving *BICRA*. In 2019, trio whole-exome sequencing (Baylor Genetics, Accession #D810045812) identified a heterozygous *BICRA* frameshift variant, c.2479_2480delinsA (p.Ala827Thrfs*15). This variant was confirmed to be de novo and was initially classified as VUS. The individual was subsequently reported as one of the original 12 patients in the discovery cohort described by Barish et al. (2020) [4], in which heterozygous pathogenic variants in *BICRA* were first established as the cause of Coffin–Siris syndrome 12 (CSS12; OMIM #619325).

Although the original report (12 April 2019) and a first addendum (26 June 2020) initially classified this as a VUS, an updated reanalysis on 13 October 2025, reclassified it as pathogenic, consistent with the diagnosis. The updated report also identified a heterozygous missense variant in *COL1A1* (c.1882G>A, p.Ala628Thr). Pathogenic variants in *COL1A1* account for approximately 90% of osteogenesis imperfecta (OI) cases [9] and have also been implicated in rare forms of Ehlers-Danlos syndrome (EDS), including classical EDS, arthrochalasia EDS, and *COL1*-related overlap disorders [10]. However, this specific variant was predicted to be tolerated/benign by in silico tools (SIFT/PolyPhen-2) and was inherited from the clinically unaffected father. Moreover, there are no published reports linking this specific variant to pathogenic connective tissue phenotypes. Accordingly, it was not considered causative of the patient’s clinical features. No additional variants in genes associated with connective tissue disorder were identified in the updated WES analysis.

**Figure 1:**
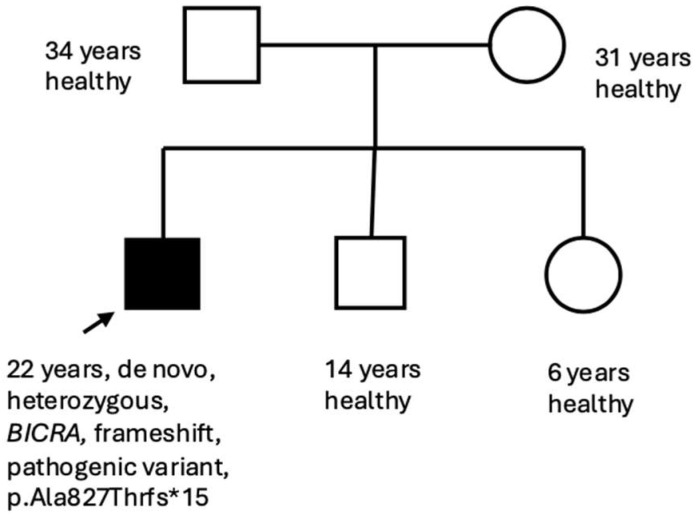
Family Pedigree. Squares indicate males and circles indicate females. Filled symbols represent affected individuals, and open symbols represent clinically unaffected individuals. The proband (arrow, filled square) carries the de novo pathogenic *BICRA* variant c.2479_2480delinsA (p.Ala827Thrfs*15). Open symbols represent the clinically unaffected parents and siblings.

**Figure 2:**
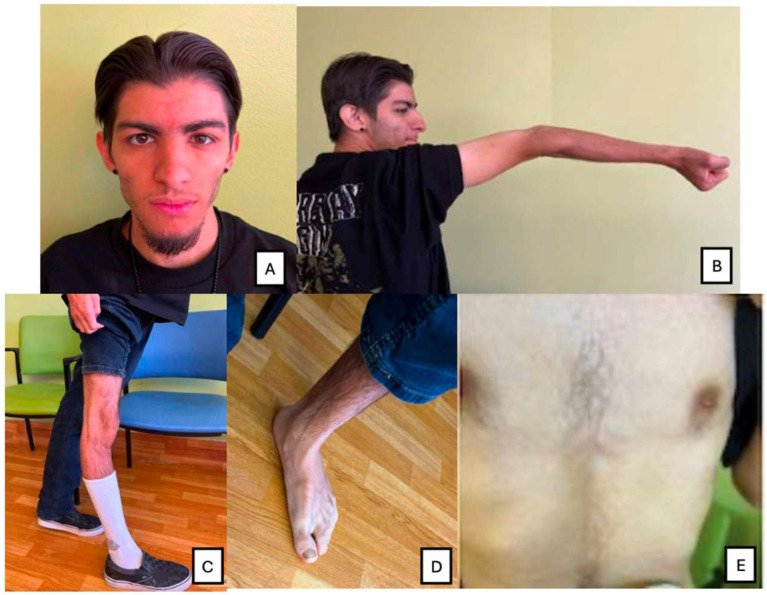
Phenotypes: (**A**) coarse face, thick hair; (**B**) hyperextended elbow; (**C**) hyperextended knees; (**D**) Flat feet; (**E**) mild pectus excavatum.

At his most recent follow-up on 16 October 2025 (22 years old), bowel function remained stable post-colectomy, arthritis was well controlled, and no further pulmonary events had occurred. He is employed in a supported work program and continues regular multidisciplinary follow-up with genetics, gastroenterology, pulmonology, and rheumatology. Family history was non-contributory, with no similar features or developmental disorders among relatives (see Figure 1). On examination at the most recent visit, the patient appeared alert and socially engaged. Growth parameters were below average (height 158 cm, −2 SD; weight 48 kg, −1.5 SD; head circumference 52 cm, −2.5 SD). Craniofacial features included brachycephaly, coarse facial features, thick eyebrows, long eyelashes, flattened nasal bridge, and a wide mouth with full lips. Hair was thick and coarse with generalized hypertrichosis. Musculoskeletal findings included joint hypermobility, mild pectus excavatum, thoracolumbar scoliosis, and hypotonia (see Figure 2). Neurological exam revealed normal gait and coordination. Brain MRI was normal. Echocardiography showed mild right-ventricular (RV) dilation with preserved systolic function. Chest CT after pneumothorax demonstrated bilateral apical bullae with small residual blebs but no active air leaks. Abdominal ultrasound confirmed resolution of the prior portal-vein thrombosis. Endocrine and metabolic studies were otherwise normal.

## 3. Discussion

### 3.1. Clinical Features and Pathogenesis

Coffin–Siris syndrome 12 (CSS12; OMIM #619325) is an autosomal dominant disorder caused by heterozygous pathogenic variants in *BICRA*, which encodes a scaffold protein of the non-canonical BAF (ncBAF) chromatin-remodeling complex. The ncBAF complex interacts with BRD9 and BRD4 to regulate enhancer accessibility and transcriptional elongation during neurodevelopment [5], and BICRA is a defining member of this complex. Functional studies confirm that BICRA physically binds other ncBAF members and is essential for complex stability. Loss of BICRA function destabilizes the ncBAF complex leading to global dysregulation of developmental gene programs. Typical clinical features include global developmental delay or mild-to-moderate intellectual disability, hypotonia, coarse facial features, hypertrichosis, and variable multisystem involvement. Additional findings may include autism spectrum disorder, behavioral abnormalities, gastrointestinal and ophthalmologic symptoms, growth retardation, and craniofacial dysmorphism [4,6,11]. Adult cohort studies indicate that visual impairment, scoliosis, and behavioral anomalies may become more prevalent over time, and cognitive outcomes range from profound intellectual disability to low-normal IQ [8]. While the role of ncBAF in neurodevelopment is well-established, its function in other tissue types is less defined. We hypothesize that *BICRA* haploinsufficiency may also disrupt transcriptional programs required for the maintenance of smooth muscle or connective tissue integrity. This potential mechanism could explain the overlapping features of hypotonia, joint laxity, visceral dysmotility, and pulmonary findings observed in our patient, although functional studies are needed to confirm this link.

### 3.2. Phenotype Comparison

The present case of a 22-year-old male with a de novo pathogenic *BICRA* frameshift variant expands the clinical spectrum of CSS12. While published cases consistently report developmental delay, intellectual disability, coarse facial features, hypotonia, and hypertrichosis as core findings [4,6,7,8], the severity and breadth of visceral and connective-tissue involvement in this individual are striking (see Table 1).

Gastrointestinal dysmotility in this case was profound, progressing from severe constipation in infancy to megacolon and recurrent bowel obstruction, ultimately requiring total colectomy.

While mild gastrointestinal symptoms such as feeding difficulties and constipation have been reported in BICRA-related CSS12 [4,6,7], the severe dysmotility requiring surgical intervention in this patient represents a rare and severe expansion of the phenotype rather than a typical feature.

This finding underscores the importance of anticipating severe gastrointestinal complications in affected individuals. Multisystem involvement further distinguishes this case. The patient developed growth hormone deficiency, short stature, generalized joint hypermobility, recurrent knee dislocations, and psoriatic arthritis. While joint laxity and short stature have been occasionally reported, autoimmune manifestations such as psoriatic arthritis are novel within the BICRA spectrum. However, it remains unclear whether this is a direct consequence of immune dysregulation related to chromatin remodeling or a coincidental comorbidity, given that autoimmune conditions are not a classic feature of CSS. Additionally, spontaneous pneumothoraces due to bullous lung disease and portal vein thrombosis represent previously unreported features, as pulmonary and vascular complications have not been systematically described in published cohorts including adult patients [8,12]; the underlying mechanism remains unknown.

Long-term follow-up provides valuable insight into the evolving natural history and management of CSS12. The patient remains clinically stable, with improved gastrointestinal function after colectomy, well-controlled arthritis, and no recurrence of pulmonary events. This case reinforces the significant phenotypic heterogeneity of *BICRA*-related CSS12 and highlights the importance of broad, multidisciplinary surveillance. Furthermore, the absence of classic digital anomalies, often seen in other SWI/SNF-related disorders, further supports the concept of gene-specific variability within this group. Expanding the recognized spectrum to include these rare visceral, pulmonary, vascular, and autoimmune complications will aid in earlier diagnostic recognition and inform anticipatory clinical management [3,13]

### 3.3. Genetic Insights

Most reported pathogenic *BICRA* variants are de novo loss-of-function (LoF) changes—typically frameshift or nonsense variants—resulting in haploinsufficiency of the ncBAF complex and disruption of chromatin remodeling presenting a characteristic but variable clinical spectrum [4,12]. Truncating *BICRA* variants (frameshift, nonsense) are associated with more severe multisystem involvement, including growth impairments, connective tissue findings, and visceral complications [6]. This patient carries a heterozygous de novo frameshift variant in *BICRA* (c.2479_2480delinsA, p.Ala827Thrfs*15), which truncates the coiled-coil domain critical for interaction with BRD9 and BRD4—key components of the non-canonical BAF (ncBAF) chromatin-remodeling complex. This variant pattern supports a genotype–phenotype correlation in which truncating mutations, as seen in this case, are associated with more severe or multisystemic manifestations. Missense variants in *BICRA*, particularly those affecting the BRD9-binding domain, may be associated with milder or atypical phenotypes, though data remain limited due to the rarity of such cases. The overall spectrum of CSS12 is variable, and long-term follow-up suggests that additional features such as joint hypermobility, autoimmune disease, and vascular complications may emerge. The clinical spectrum is broad, and ongoing surveillance is recommended to identify late-manifesting complications.

### 3.4. Management

Management of BICRA-related Coffin–Siris syndrome 12 (CSS12) is supportive and multidisciplinary, targeting neurodevelopmental, gastrointestinal, musculoskeletal, and behavioral complications [2,8,12]. In this case, severe intestinal dysmotility required total colectomy, psoriatic arthritis responded to adalimumab, and pulmonary and vascular complications were successfully managed with monitoring and anticoagulation. No targeted therapy currently exists, but experimental modulation of BET-protein interactions (BRD4/BRD9) has been proposed as a future approach [14,15]. Regular surveillance for late-manifesting complications, including visual impairment, scoliosis, obesity, behavioral anomalies, and organ system involvement, particularly rare but severe visceral and vascular complications and coordinated care across specialties, remain essential to optimize long-term outcomes [12].

## 4. Conclusions

This report provides the first detailed longitudinal documentation of a patient with *BICRA*-related Coffin–Siris syndrome 12 extending into adulthood, demonstrating that the disorder’s manifestations extend far beyond neurodevelopmental delay. The patient’s presentation of severe intestinal dysmotility requiring colectomy, recurrent spontaneous pneumothoraces due to bullous lung disease, and vascular thrombosis represents a rare and expanded phenotype. These findings suggest that BICRA haploinsufficiency may impact organ systems beyond the central nervous system. Recognition of these potential complications is clinically important for early surveillance and multidisciplinary management. Continued collection of longitudinal data will further refine genotype–phenotype correlations and improve prognostic counseling for affected families.

## Figures and Tables

**Table 1:** CSS12 phenotypes comparison with selected publications **.

Features/Mutation	22-Year-Old Male (Current Case)	Asadauskaite et al., 2023 [6]	Tu et al., 2023 [7]	Barish et al., 2020 [4]	Schrier et al., 2025 [12]
***BICRA* Variant**	c.2479_2480delinsA (p.Ala827Thrfs*15), de novo, *frameshift*	c.535C>T (p.Gln179), de novo, nonsense	c.1666C>T (p.Gln556*), de novo, nonsense	10 LoF *, 2 missense (various exons)	Multiple LoF *, missense
**Developmental Delay**	Global, walked at 18 months, mild-moderate ID (IQ ≈ 75)	Global, microcephaly, moderate ID	Language delay, mild ID	Moderate-severe DD/ID, autism, behavioral	Moderate-severe DD/ID
**Facial Features**	Brachycephaly, thick lips, wide mouth, coarse hair, hypertrichosis	Craniofacial dysmorphism, coarse features	Mild dysmorphism	Coarse features, thick lips, wide mouth	Coarse features, hypertrichosis
**Hypotonia**	Present since infancy	Present	Present	Present	Present
**Gastrointestinal**	Severe constipation, megacolon, recurrent obstruction, total colectomy	Severe constipation, megacolon, feeding diff	Mild GI features	Feeding difficulties, constipation (variable)	Feeding difficulties, constipation
**Growth**	Short stature, GH deficiency	Low birth weight, growth retardation	Not reported	Short stature (variable)	Short stature
**Skeletal/Connective Tissue**	Joint hypermobility, recurrent knee dislocations, psoriatic arthritis	Not reported	Not reported	Variable, some with joint laxity	Variable
**Other Visceral Involvement**	Spontaneous pneumothoraces, bullous lung disease, portal vein thrombosis, RV dilation	Urinary tract impairment, visual impairment	Not reported	Rare, not systematically reported	Not well described
**Ophthalmologic**	Not reported	Visual impairment	Not reported	Variable	Variable
**Behavioral/Autism**	Mild-moderate ID, no autism reported	Moderate ID	Mild ID	Autism, behavioral challenges	Autism, behavioral challenges
**Long-term Follow-up**	Stable, improved GI post-colectomy, controlled arthritis, no pulmonary recurrence	Not reported	Not reported	Limited long-term data	Limited long-term data

* LoF: loss of function. ** Publications were selected for inclusion based on the availability of detailed clinical data regarding systemic manifestations (particularly gastrointestinal, growth, and skeletal features) to allow for a meaningful comparison of the expanded phenotype.

## Data Availability

The original contributions presented in the study are included in the article, further inquiries can be directed to the corresponding author.
